# Isolation, identification, and pathogenicity of a ALV-K strain from Chinese indigenous chicken breed

**DOI:** 10.1016/j.psj.2022.102116

**Published:** 2022-08-05

**Authors:** Hao Chen, Youjiang Diao, Xiaolong Sun, Yixin Wang

**Affiliations:** ⁎ College of Agricultural Technology, Shandong Agriculture and Engineering University, Jinan, China; † Liaocheng Engineering Technology Research Center for Broiler Healthy Breeding, Liaocheng, China; ‡ College of Animal Science and Veterinary Medicine, Shandong Agricultural University, Tai'an, China

**Keywords:** subgroup K Avian leukosis virus, Chinese indigenous breed, virus isolation and identification, full-length genomic sequence analysis, pathogenicity

## Abstract

Subgroup K avian leukosis virus (**ALV-K**) is a new subgroup of avian leukosis virus (**ALV**) first identified in Chinese indigenous chickens in recent years. In this study, an ALV-K strain was isolated from Luhua chicken in Shandong province, China, and designated SD20LH01. The full-length genomic sequence of SD20LH01 was 7491 bp, which had the highest homology with ALV-K reference strains GDFX0601, GDFX0602 and GDFX0603. The nucleotide homology of *env* gene of SD20LH01 with reference strains of subgroup A, B, C, D, E, and J was ranged from 57.1 to 93.2%, while 94.1 to 99.4% with other ALV-K reference strains. The nucleotide difference of SD20LH01 mainly clustered with *gp85* gene and U3 sequence when compared with the reference strain of ALV-K. In order to investigate the pathogenicity of SD20LH01, SPF chicken embryos were infected by yolk sac inoculation, and 1-day-old chickens were infected by intraperitoneal inoculation of SD20LH01. The results showed that yolk sac inoculation of SD20LH01 could induce persistent viremia, growth retardation and reduce the immune response to NDV and AIV-H9 vaccines. However, intraperitoneal inoculation in 1-day-old chickens could only induce a low level of viremia. In addition, no tumors were found in infected chickens during the animal experiments. This study enriched the genomic sequence data of ALV-K isolated in Chinese indigenous chickens, and laid a foundation for further study on the pathogenesis and prevention of ALV-K.

## INTRODUCTION

Avian leukosis (**AL**) is the general name of a variety of avian tumor diseases caused by avian leukosis virus (**ALV**), which is one of the most common tumor diseases in poultry and widely exists in all kinds of chicken flocks in the world (Payne and Nair, 2012). In addition to causing tumors, ALV can also induce immunosuppression and growth retardation in infected chickens, which poses a great threat to the poultry industry (Feng and Zhang, 2016). According to the antigenicity of envelope protein, host range and neutralization experiment, ALV can be divided into subgroups A∼J. Among them, subgroup J ALV (**ALV-J**) can induce myeloid leucosis and hemangioma, which is the most virulent subgroup (Arshad et al., 1997).

In recent years, with the progress of ALV clearance project in China, some ALV strains had been isolated from Chinese indigenous chickens. Interestingly, the amino acid homology of Env protein between these strains and subgroup A∼E ALV were 77.7 to 84.6%, and the homology with ALV-J were less than 40%. However, the homology between these strains was more than 90%. Therefore, we believed that those strains belonged to a novel subgroup ALV, which would be named as subgroup K ALV (**ALV-K**), and its prototype strain was JS11C1 which isolated from Chinese indigenous chickens in 2012 (Cui et al., 2014; Prikryl et al., 2019). Subsequently, a series of ALV-K strains from yellow feather broilers and other Chinese indigenous chicken breeds in South China were successively isolated (Dong et al., 2015; Su et al., 2018; Liang et al., 2019). Since most ALV-K strains were isolated from clinically healthy chickens, it was speculated that ALV-K had a mild pathogenic characteristic. In addition, studies had found that ALV-K was associated with gliomas in poultry in Japan (Nakamura et al., 2011).

In Shandong Province, China, a variety and large number of indigenous chicken breeders were distributed, like Luhua chicken, Laiwu black chicken, and Bairi chicken, etc. During daily monitoring in our lab, a higher expression level of ALV p27 antigen in embryos of a Luhua chicken flock was observed. To investigate whether this flock was infected with ALV, chicken serums were collected and inoculated into DF-1 cells, and an ALV strain named SD20LH01 was successfully isolated. The results of genome sequencing indicated that SD20LH01 strain was ALV-K. In addition, specific pathogen-free chickens (**SPF**) chickens were infected by yolk sac inoculation and 1-day-old chickens were infected with SD20LH01 intraperitoneally, and pathogenicity of SD20LH01 was further explored.

## MATERIALS AND METHODS

### Virus Isolation and Identification

The DF-1 cells were inoculated into the tissue culture plate. When the DF-1 cells were grown with 70 to 80% of the plate, the culture medium was discarded and washed twice with PBS. Then, the collected chicken serum was inoculated into DF-1 cells, and cultured for another 2 h in an incubator at 37°C with 5% CO_2_. After that, fresh DMEM medium with 1% Fetal bovine serum (**FBS**) was added and maintained for 7 d, and then blind passaged 3 times. Finally, ALV p27 antigen was detected with avian leukosis virus antigen kit (IDVET, France), and ALV *gp85* gene was amplified and sequenced to identify the subgroup. To exclude contamination of other pathogens, Reticuloendotheliosis virus (**REV**), Marek's disease virus (**MDV**), avian reovirus (**REO**) and Fowl adenovirus (**FAdV**) were detected by PCR or RT-PCR. The sequence of those primers were listed in Table 1. The p27-positive cellular supernatants were collected as viral stocks and stored at −80°C, and this isolated strain was designated SD20LH01.

**Table 1:** Primers used in this study.

NO.	Primer	Sequence (5’-3’)	Length/bp
1	MDV-F	GCCTTTTATACACAAGAGCCGAG	560
	MDV-R	TTTATCGCGGTTGTGGGTCATG	
2	REO-F	AATCCCTTGTTCGTCGATGCT	1015
	REO-R	AAATTGGAGATGGCAGTGGA	
3	FAdV-F	AATTTCGACCCCATGACGCGCCAGG	508
	FAdV-R	TGGCGAAAGGCGTACGGAAGTAAGC	
4	REV-F	TATAATGTGGGAGGGAGC	550
	REV-R	GCCCCCAAATGTTGTA	
5	Env-F	GGATGAGGTGACTAAGAAAG ALV(all)-R (5’- ACACTACATTTCCCCCTCCCTAT-3’)	2200
	Env-R	ACACTACATTTCCCCCTCCCTAT	
6	ALV-F1	CACCACATTGGTGTGCACCTGGGT	2566
	ALV-R1	GAAGGGGCCACTGGTCAATCCACA	
7	ALV-F2	GAGATTGTCTGCAGGGCCTAGGGCT	2738
	ALV-R2	TGGCAGCAAGGGTGTCTTCTCCG	
8	ALV-F3	GAGGTGACTAAGAAAGATGAGGCGA	2218
	ALV-R3	CATCTCCCCCTCCCTATGCGAAAGC	
9	ALV-F4	GGCTTCGGTTGTACGCGGATAGGA	556
	ALV-R4	CTTCCAACGACCCTCTGAGTGCTCG	

Note: Primers 1-4 were used to detect the possible contamination of MDV, REO, FAdV and REV in the isolated strains, primer 5 was used to amplify the *env* sequence of all subgroups of ALV, and primers 6-9 were used to amplify the whole gene sequence of the isolated strains.

### Indirect Immunofluorescence Assay (IFA)

The sequencing results of *gp85* gene revealed that SD20LH01 was belonged to ALV-K. To further verify the isolated virus, immunofluorescence assay (**IFA**) experiments were performed. The cell supernatant was discarded, and infected DF-1 cells were fixed with paraformaldehyde at 4°C overnight. After that, permeabilized with 0.25%Triton X-100 for 15 min at room temperature. Then, cells were washed 3 times with PBS and incubated with mouse anti ALV-K gp85 polyclonal antibody for 1 h at 37°C. Next, a FITC-conjugated goat anti-mouse antibody (Abcam, UK) was used as the secondary antibody for 1 h at 37°C. Finally, the cells were washed 3 times again with PBS and observed under a fluorescence microscope. The mouse anti ALV-K gp85 polyclonal antibody was prepared in our laboratory. Briefly, ALV-K gp85 gene of JS11C1 strain was amplified and inserted into pET32-a (+) prokaryotic expression vector, and transformed into BL21 *E. coli* cells to obtain the recombinant protein. Mouse were immunized with the purified recombinant protein mixed with Freund's complete adjuvant to prepare the anti-ALV-K gp85 serum.

### Full-Length Genome Sequencing of SD20LH01

According to the ALV genome sequence published in GenBank, 4 pairs of primers were designed using Snapgene software and synthesized by Shanghai Sangon company to amplify the full-length sequence of SD20LH01. The sequence primers used in this study were shown in Table 1. DNA of DF-1 cells infected with SD20LH01 was extracted and the full-length sequence of SD20LH01 was amplified by PCR. The PCR reaction system was as following: 2 × Ex Taq Mix 12.5 μL (Takara, Dalian, China), upstream primer 1 μL, downstream primer 1 μL, DNA 2 μL, and ddH2O 8.5 μL. The reaction procedure was as follows: pre-denaturation at 94 °C for 5 min, denaturation at 94°C for 30 s, annealing at 60°C for 30s, extension at 72°C for 2 min, with a total of 35 cycles, and extension at 72°C for another 10 min and preservation at 4°C. The PCR products were purified and sequenced by Shanghai Sangon company.

### Analysis of SD20LH01 Provirus Sequence

The whole genome sequences of ALV reference strains were downloaded from Genebank, and the Clustal W algorithm was used to calculate the homology of ALV reference strains and SD20LH01. In addition, the phylogenetic tree analysis was carried out based on the whole genome and U3 sequence of ALV using Mega 7.0 software by neighbor joining method. The information of ALV reference strains selected in this study was shown in Table 2.

**Table 2:** Information of avian leukosis virus (ALV) reference strains used for sequence alignment.

Strains	Subgroup	Origin	GenBank NO.
SDAU09C3	A	China	HM452340
MAV-1	A	France	L10922
MAV-2	B	France	L10924
SDAU09C2	B	China	HM446005
Prague C	C	USA	J02342
RSV- Schmidt-Ruppin D	D	Japan	D10652
ev-1	E	USA	AY013303
SD0501	E	China	EF467236
HPRS103	J	UK	Z46390
NX0101	J	China	DQ115805
JS11C1	K	China	KF746200
JS14CZ01	K	China	KY490695
JS14CZ02	K	China	KY490696
GDFX0601	K	China	KP686142
GDFX0602	K	China	KP686143
GDFX0603	K	China	KP686144
TW-3593	K	China	HM582658
HB2015032	K	China	KY581580
GD14LZ	K	China	KU605774

### Determination of Virus Titer and Replication Dynamics

The virus titer (median tissue culture infective dose, TCID_50_) of SD20LH01 was quantified using Karber method according to the references. SD20LH01, ALV-K strain JS11C1, ALV-A strain SDAU14A1, and ALV-J strain NX0101 were inoculated into DF-1 cells with 10^3.5^ TCID_50_ respectively at the same time. Then, the cellular supernatants were collected at 12 h, 24 h, 48 h, 96 h, and 144 h postinfection. ALV-p27 antigen were detected by detected with avian leukosis virus antigen kit (IDVET, France), and the replication dynamics of different strains were obtained with Graphpad prism 9.0 software.

### Animal Experiment Design

To investigate the pathogenicity of SD20LH01, the animal experiments were performed. The animal experiments were approved by Shandong agricultural university animal care and use committee. Care and maintenance of all chickens were in accordance with the guidelines of the Committee on the Ethics of Animal of Shandong Agricultural University and the biosecurity guidelines. 80 leghorn specific pathogen-free chicken (**SPF**) embryos (SAIS Poultry Co., Jinan, China) were divided into four groups randomly (20 in each group), and SD20LH01 was inoculated by yolk sac at 6th embryo age and intraperitoneally at 1-day-old post-hatch respectively, chickens inoculated intraperitoneally with NX0101, a ALV-J strain, at 1-day-old post-hatch were served as positive control, and chickens injected with PBS buffer at 1-day-old post-hatch were served as negative control (Table 3). Chicken serum in each group collected at 1 d, 1, 3, 5, 8, 14, 17, 20, and 22 wk were inoculated into DF-1 to detect ALV viremia. To determine the inhibitory effect of SD20LH01 on chicken body weight, the body weights of all chickens were weighed and recorded at wk 1, 2, 3, 4, 5, 6 post-hatch.

**Table 3:** Grouping of animal experiment.

Group	Treatment	Numbers
1	Injected with 10^3^ TCID_50_ SD20LH01 by yolk sac at 6th embryo age	20
2	Injected with 10^3^ TCID_50_ SD20LH01 intraperitoneally at 1-day-old post-hatch	20
3	Injected with 10^3^ TCID_50_ NX0101 intraperitoneally at 1-day-old post-hatch	20
4	Injected with PBS buffer intraperitoneally at 1-day-old post-hatch	20

### Determination of Antibody Responses to NDV and H9-AIV Vaccination

To investigate the immunosuppressive effects of SD20LH01 on response to NDV and H9-AIV vaccination, chickens in all groups were vaccinated subcutaneously with inactivated NDV (Qilu Animal Health Products Co., Jinan, China) and AIV-H9 (Qilu Animal Health Products Co.) at 1-wk post-hatch. NDV and H9-AIV titers were measured in serums collected at wk 3, 4, 5 post-hatch with the hemagglutination inhibition (**HI**) assay.

### Data Statistical Analysis

All the results are presented as the Means ± SEMs. All statistical analyses were performed using SPSS statistical software package for Windows, version 17.0 (SPSS Inc., Chicago, IL). Duncan's multiple-range test was performed to determine the differences of body weight, bursa and thymus index, and antibody response to NDV and AIV-H9 between different groups.

## RESULTS

### Virus Isolation and Identification in DF-1 Cells

**Figure 1:**
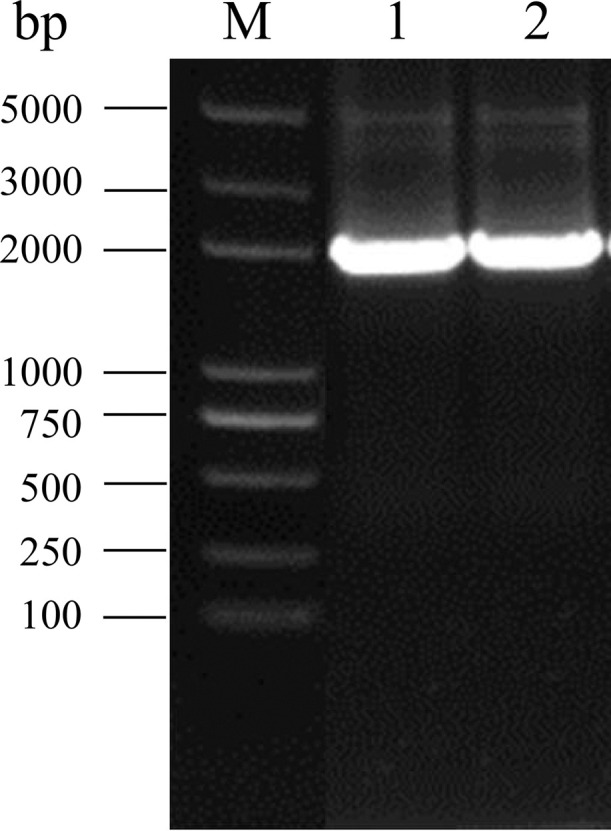
Electrophoresis of *env* sequence of isolated strains. M: 5 000 DL Marker; 1, 2: Amplification of *env* gene of isolated strains.

**Figure 2:**
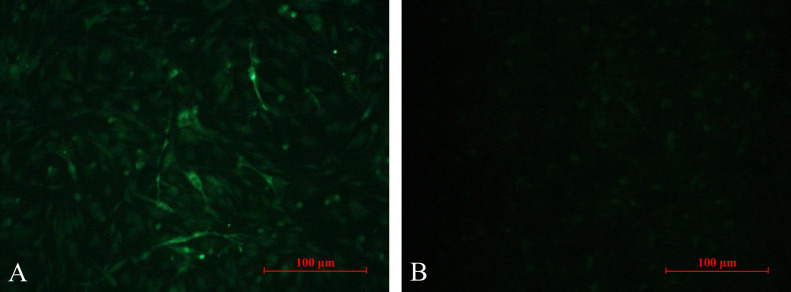
IFA results of DF-1 cells infected with SD20LH01. A. DF-1 cells infected with SD20LH01; B. Uninfected DF-1 cells (400 ×).

Chicken serums were inoculated into DF-1 cells and passaged for three generations, and ALV p27 antigen was detected using commercial ELISA kit in the cellular supernatant. The results showed that 10 (10/27) cell supernatants were p27-positive. Then, *env* gene was amplified and sequenced for further analyze (Figure 1). The results showed that the homology of *env* gene of those 10 isolated viruses was more than 99.5%, indicating that a same viral strain was isolated from this chicken flock. Therefore, the isolated strain was designated SD20LH01. In addition, the homology of *env* gene between SD20LH01 and ALV-K reference strains was ranged from 94.1 to 99.4%, indicating that SD20LH01 was belonged to subgroup K ALV. The results of PCR or RT-PCR demonstrated that the viral stock did not contain REV, MDV, REO and FAdV contamination. To further confirm the isolation of SD20LH01, IFA was detected using ALV-K gp85 specific polyclonal antibody. The results showed that the cytoplasm of infected DF-1 cells showed green positive fluorescence staining, while there was no fluorescence signal in uninfected DF-1 cells (Figure 2).

### Sequencing and Genetic Characterization of SD20LH01 Proviral Genome

**Figure 3:**
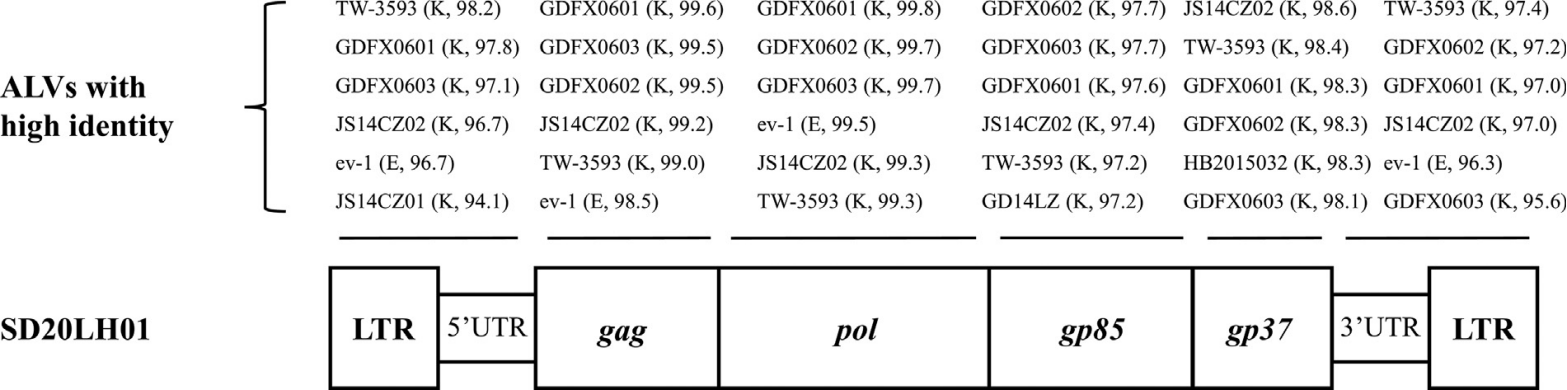
The genomic structure of SD20LH01. The reference strains with high sequence similarity to this isolate are shown above the genome structure.

DNA of DF-1 cells infected SD20LH01 was extracted, and the ALV proviral genome was amplified by PCR. Then, purified PCR products were sequenced and the full-length genomic sequence of SD20LH01 was obtained. The full-length SD20LH01 provirus sequence was 7,491 bp, which had a typical genome structure of replication complete retrovirus. The genomic sequence of SD20LH01 had been submitted to GenBank with the accession number OP035379. It encoded *gag, pol* and *env* genes in turn, and the 2 ends were completely consistent LTR sequences. *Gag* gene was located in 559 to 2,664 (2,106 bp), *pol* gene was located in 2,679 to 5,369 (2,677 bp), and *env* gene was located in 5,233 to 7,047 (1,791 bp). The length of LTR at both ends was 280 bp, which is located in 1 to 280 and 7,214∼7,491 of the genome, respectively, which consisted of U3, R, and U5, with lengths of 179, 23, and 78 bp, respectively. The homology of full-length genomic sequence of SD20LH01 and other ALV-K reference strain was ranged from 96.65 to 99.0% (Figure 3), with the highest homology with GDFX0601 (99.0%), and relatively low homology with JS11C1 (96.65%).

### Analysis of SD20LH01 Coding Sequences

**Figure 4:**
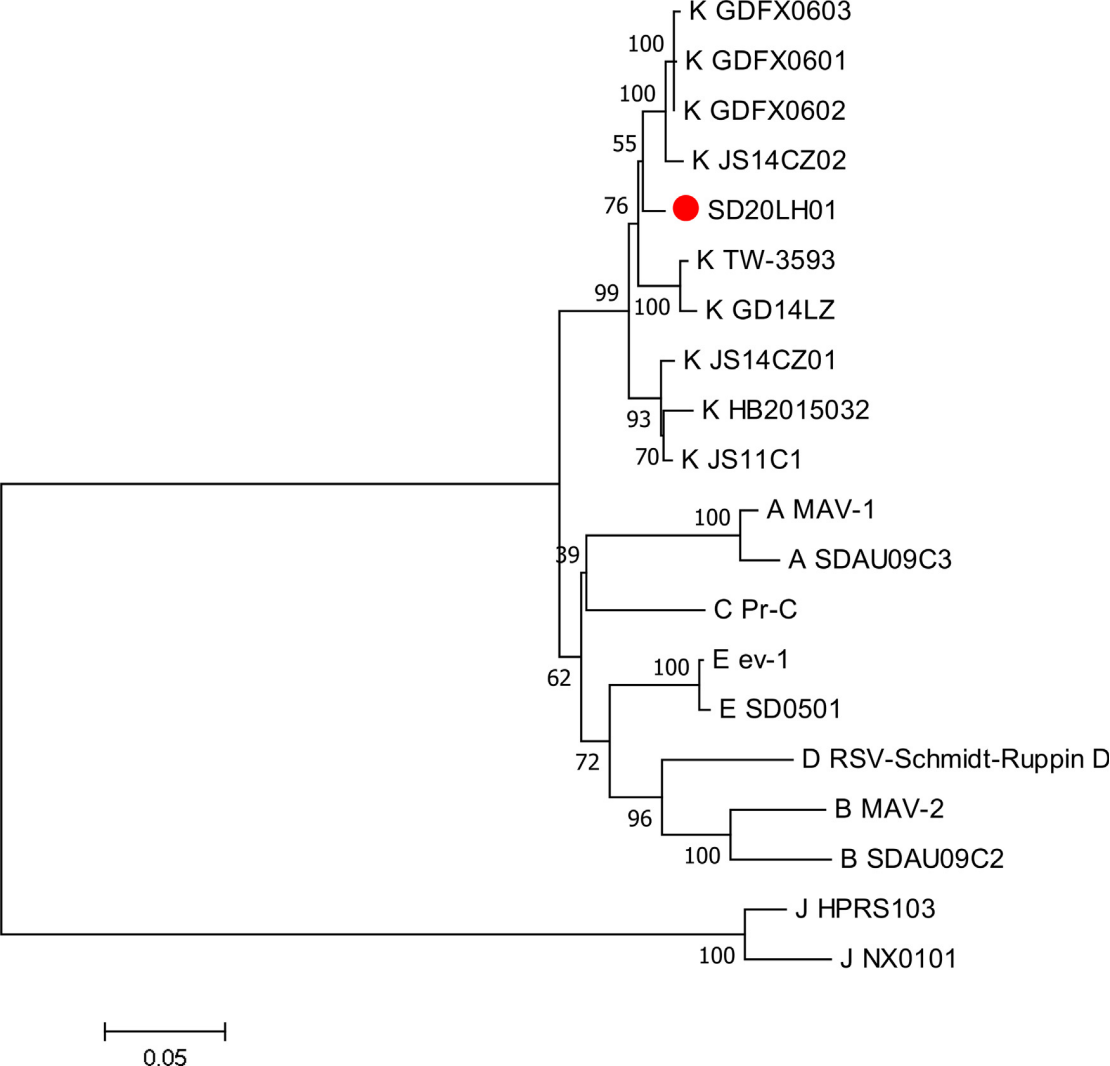
Phylogenetic tree based on *gp85* gene of SD20LH01 and reference strains. The phylogenetic analysis was carried out by Neighbor-joining method by a bootstrap analysis of 1,000 replicates using MEGA 7.0 software program. SD20LH01 was labeled with red circles.

The homology analysis of the coding regions showed that the *gag* and *pol* genes of SD20LH01 were relatively conservative, and there was no significant variation when compared SD20LH01 with other reference strains. The nucleotide sequence of *gag* gene of SD20LH01 had a 93.8 to 99.1% homology with the reference strains, and the nucleotide sequence of *pol* gene had a 97.4 to 99.7% homology with the reference strain. Envelop protein encoded by *gp85* gene was closely related to the neutralizing antibody induced by ALV. The nucleotide sequence of *gp85* gene of SD20LH01 had a 39.9 to 87.5% homology with the reference strain of subgroup A, B, C, D, E and J, and 95.3 to 97.8% homology with the reference strain of subgroup K. The phylogenetic analysis based on *gp85* gene was basically consistent with the homology comparison that SD20LH01 was located in the same branch with ALV-K reference strains JS14CZ02, GDFX0601, GDFX0602, and GDFX0603, while had a distant genetic relationship with other subgroups ALV (Figure 4).

### Analysis of SD20LH01 LTR Sequences

**Figure 5:**
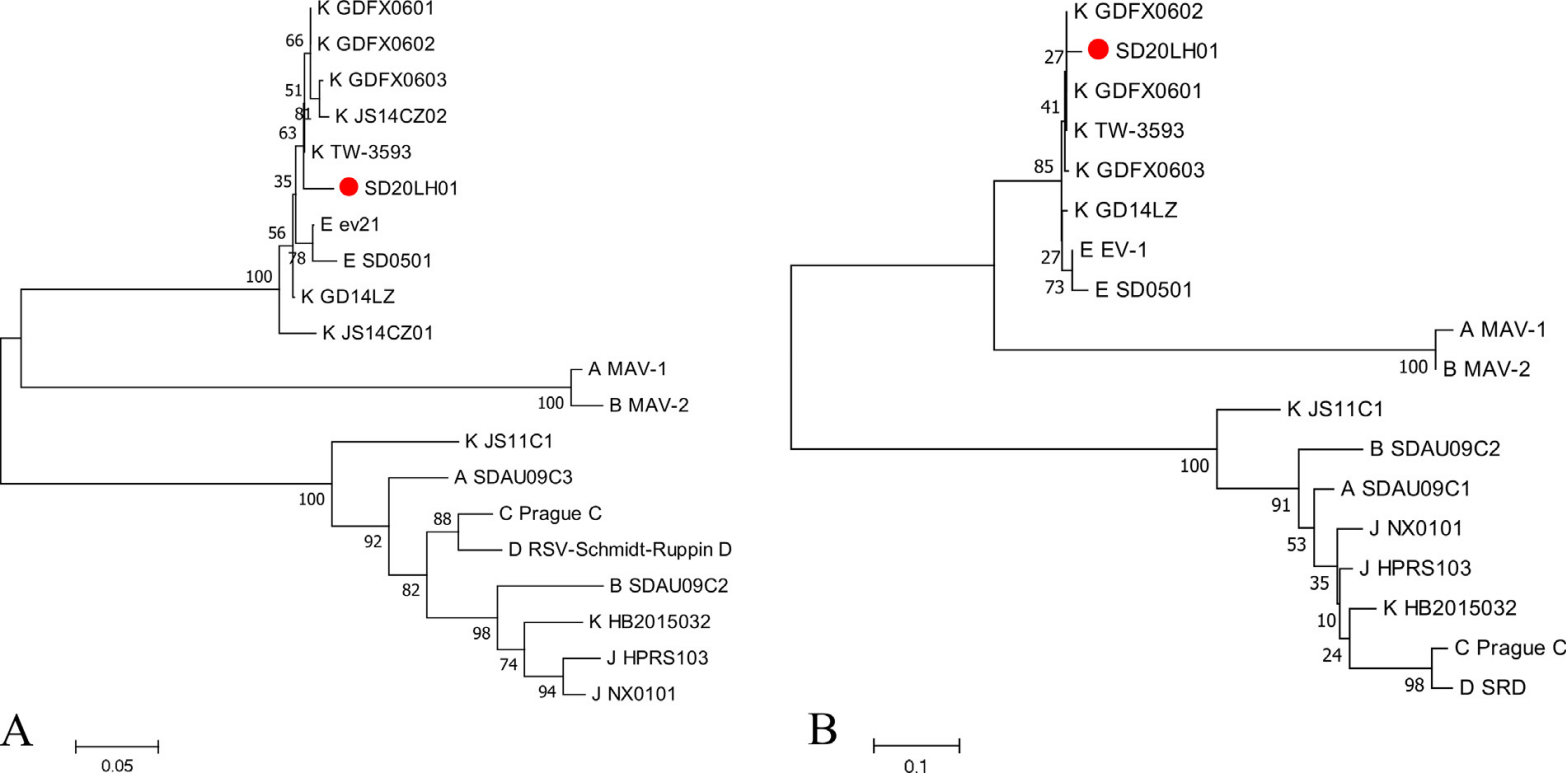
Phylogenetic tree based on LTR (A) and U3 sequence (B) of SD20LH01 and reference strains. The phylogenetic analysis was carried out by Neighbor-joining method by a bootstrap analysis of 1,000 replicates using MEGA 7.0 software program. SD20LH01 was labelled with red circles.

LTR gene consisted of U3, R, and U5 regions, among which U3 was the most variable region. The homology of LTR sequence between SD20LH01 and reference strains was ranged from 58.9 to 98.2%, with the highest TW-3593 (98.2%). As shown in the genetic evolution tree based on LTR sequence, SD20LH01 was on the same branch with TW-3593, GDFX0601, and GDFX0602. The homology of U3 region between SD20LH01 and reference strains was ranged from 43.8 to 98.3%, with the highest GDFX0601 and GDFX0602 (98.3%), and the genetic evolution analysis also confirmed this result (Figure 5). The homology of U5 region between SD20LH01 and reference strains was 84.6 to 98.7%, with the highest JS11C1, JS14CZ01, and GD14LZ (98.7%).

### Determination of Viral Replicate Kinetics in DF-1 Cells

**Figure 6:**
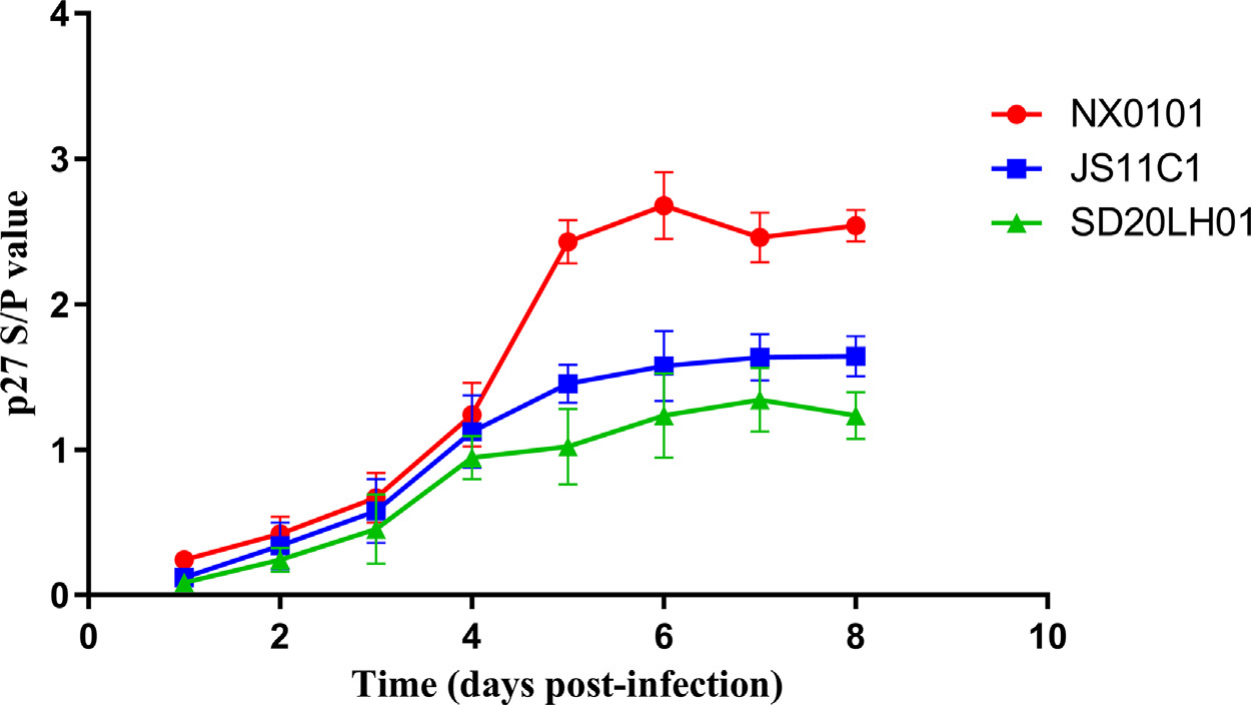
Comparison of viral replication dynamics of three viruses.

DF-1 cells were inoculated with quantified virus ALV-J NX0101, ALV-K prototype strain JS11C1, and SD20LH01 isolated in this study. The replicate kinetics were determined using the cellular supernatants by ELISA. As shown in Figure 6, the trend of virus replication was significantly increased with the prolong of maintenance time. Among them, ALV-J NX0101 strain had the strongest replication ability, while JS11C1 strain has the second, and SD20LH01 strain had the weakest replication ability.

### Dynamics of Viremia in SD20LH01 Infected SPF Chickens

SPF chickens were injected with SD20LH01 by yolk sac inoculation at 5th embryo age and intraperitoneal inoculation at 1-day-old post-hatch respectively. Dynamic of viremia in 7 wk postinfection was listed as Table 4. As shown in Table 4, chickens in control group were negative for viremia throughout the experiment. The results showed that almost all the chickens infected with SD20LH01 by yolk sac from d 1 to wk 7, while viremia occurred in 50% chickens infected with SD20LH01 intraperitoneally. As a positive control, ALV-J infected chickens manifested a high rate of viremia.

**Table 4:** Positive rates of viremia in chickens at different time points.

Group	1 d	1 wk	2 wk	3 wk	4 wk	5 wk	6 wk	7 wk
Yolk sac	90% (18/20)	90% (18/20)	90% (19/20)	90% (18/20)	85% (17/20)	85% (17/20)	90% (18/20)	90% (18/20)
Intraperitoneally	0% (0/20)	0% (0/20)	30% (6/20)	40% (8/20)	40% (8/20)	50% (10/20)	50% (10/20)	45% (9/20)
ALV-J control	0% (0/20)	40% (8/20)	84.2% (16/19)	83.3% (15/18)	88.8% (16/18)	72.2% (13/18)	72.2% (13/18)	88.8% (16/18)
Uninfected control	0% (0/20)	0% (0/20)	0% (0/20)	0% (0/20)	0% (0/20)	0% (0/20)	0% (0/20)	0% (0/20)

### Influence of SD20LH01 Infection on Body Weight

**Figure 7:**
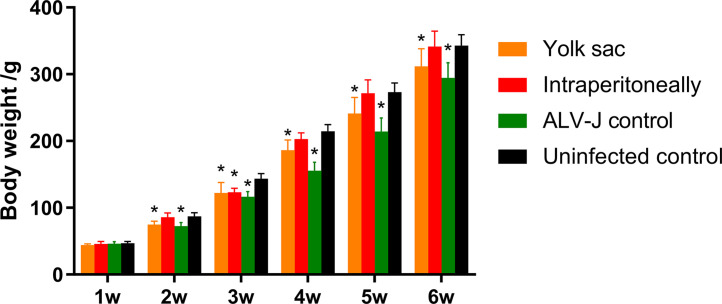
Influence of SD20LH01 infection on the body weights in SPF chickens. * indicates significant differences (*P* < 0.05), based on Duncan's multiple range test. The error bars represent the SEM.

Body weight of all chickens in wk 1 to 6 post-hatch were determined to evaluate the inhibitory effect of SD20LH01 infection on production performance. The results showed that there was no difference between intraperitoneally infected group and control group in most time points other than wk 3 (Table 5, Figure 7). However, infection of SD20LH01 by yolk sac could exert a significant influence on body weight of SPF chickens. In addition, as a positive control, there was a significant difference of body weight between ALV-J infected chickens and control group.

**Table 5:** Influence of SD20LH01 infection on body weight.

Group	1 wk	2 wk	3 wk	4 wk	5 wk	6wk
Yolk sac	44.1 ± 1.7 a	74.9 ± 4.9 a	122.2 ± 15.6 a	186.1 ± 15.3 a	241.3 ± 23.7 a	311.8 ± 26.3 a
Intraperitoneally	45.8 ± 3.8 a	85.9 ± 6.2 b	123.3 ± 5.7 a	202.8 ± 9.3 b	271.5 ± 19.9 b	341.6 ± 22.9 b
ALV-J control	46.3 ± 2.7 a	72.6 ± 5.1 a	116.4 ± 7.6^a^	155.4 ± 12.6^c^	214.1 ± 20.4 c	294.6 ± 22.4 c
Uninfected control	46.7 ± 2.8 a	87.2 ± 5.4 b	143.6 ± 7.5 b	214.5 ± 9.9 b	273.1 ± 13.7 b	342.8 ± 16.3 b

abc Different lowercase means significant difference (*P* < 0.05), and the same lowercase means no obvious difference (*P* > 0.05).

### Influence of SD20LH01 Infection on Humoral Immune Response to NDV and AIV-H9 Vaccination

**Figure 8:**
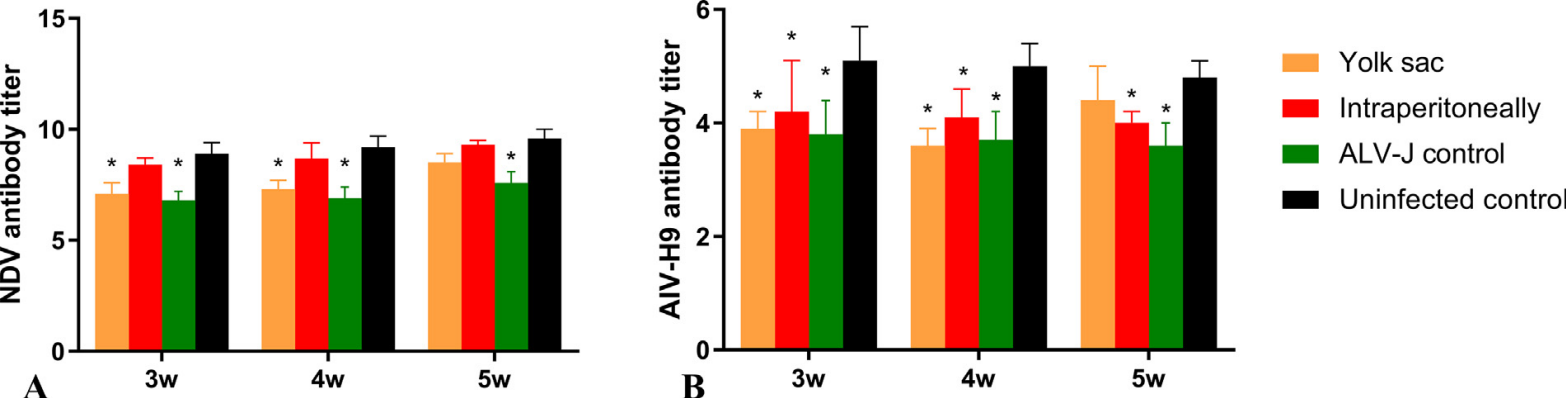
The effect of SD20LH01 infection on the immune responses against NDV and AIV-H9 vaccines in SPF chickens. * indicates significant differences (*P* < 0.05), based on Duncan's multiple range test. The error bars represent the SEM.

To investigate the inhibitory effect of SD20LH01 infection on humoral response to vaccination, all chickens were vaccinated with NDV and AIV-H9 inactivated vaccine, and antibody titers of NDV and AIV-H9 were determined. The results showed that there was no difference of NDV antibody titers between intraperitoneally infected chickens and control group chickens at wk 3, 4, 5 post-hatch (Table 6, Figure 8). However, NDV titers of chickens infected with SD20LH01 by yolk sac were lower than those in control group. As regard for AIV-H9, AIV-H9 titers of chickens infected with SD20LH01 both intraperitoneally and by yolk sac was significantly lower than control group at wk 3, 4, 5 post-hatch.

**Table 6:** Influence of SD20LH01 infection on NDV and AIV-H9 vaccination response.

Group	NDV titers post-vaccination			AIV-H9 titers post-vaccination		
	3 wk	4 wk	5 wk	3 wk	4 wk	5 wk
Yolk sac	7.1 ± 0.5 a	7.3 ± 0.4^a^	8.5 ± 0.4^a^	3.9 ± 0.3 a	3.6 ± 0.3 a	4.4 ± 0.6 a
Intraperitoneally	8.4 ± 0.3 b	8.7 ± 0.7^b^	9.3 ± 0.2 b	4.2 ± 0.9 a	4.1 ± 0.5 a	4.0 ± 0.2 b
ALV-J control	6.8 ± 0.4 a	6.9 ± 0.5 c	7.6 ± 0.5 c	3.8 ± 0.6 a	3.7 ± 0.5 a	3.6 ± 0.4 b
Uninfected control	8.9 ± 0.2 b	9.2 ± 0.5 b	9.6 ± 0.4^b^	5.1 ± 0.6 b	5.0 ± 0.4 b	4.8 ± 0.3 a

abc Different lowercase means significant difference (*P* < 0.05), and the same lowercase means no obvious difference (*P* > 0.05).

## DISCUSSION

ALV is an immunosuppressive pathogen which can induce tumors in all kinds of chickens. At present, there is no effective drug or vaccine available to use. Since ALV mainly transmitted vertically, strict monitoring and clearance measures are performed to prevent and control this disease. In the 1990s, ALV was basically eradicated in the breeding population in western countries. In China, ALV in most white feather broilers and layers had been basically excluded through clearance measures. However, the ALV infection rate in some Chinese indigenous breeds was still high.

ALV can be divided into different subgroups according to the viral envelope protein (Rainey et al., 2003). Among them, subgroup A/B ALV mainly causes lymphocytic tumors, while subgroup J ALV mainly induces myeloid tumors. In recent years, some unique ALV strains had been isolated in the process of ALV clearance in indigenous breed chickens in China. The *gp85* genes of those ALV isolates were relatively far away from the *gp85* gene of other known subgroups, while they had a high homology with each other (Dong et al., 2015). Therefore, they were considered to be a new subgroup and named as subgroup K ALV, and their prototype strain was JS11C1 isolated from Chinese Luhua chicken in 2012 (Cui et al., 2014). Subsequently, more ALV-K strains were isolated from yellow feather broilers and other Chinese indigenous chickens, suggesting that ALV-K might have been existed in Chinese chicken flocks for a long time. Since most ALV-Ks were isolated from clinically healthy chickens, it was generally considered that ALV-K was mild pathogenic. Limited research data also suggested that ALV-K might be associated with the occurrence of avian glioma. However, recent studies also shown that the recombinant ALV-K had an increased virulence, which was worthy of our concern (Lv et al., 2019).

In order to investigate the ALV infection status in a Chinese indigenous chicken flock in Shandong Province, we collected chicken eggs and detected p27 antigen using commercial ELISA kit. The results showed that the positive rate of p27 antigen in eggs was 21%, indicating the existence of ALV infection in this flock. In this study, chicken serums were inoculated into DF-1 cells and then passaged for three generations, and a ALV strain named SD20LH01 was successfully isolated. To confirm the subgroup of SD20LH01, *env* genes were amplified and sequenced. The nucleotide homology of *gp85* gene of SD20LH01 with the reference strain of subgroup K ALV was 94.1 to 99.4%, and the genetic evolutionary analysis based on *gp85* gene also suggests that SD20LH01 belonged to subgroup K. PCR or RT-PCR were also performed to detected other common pathogens like REV, MDV, REO, and FAdV in the cellular supernatant, and it confirmed that no viral contamination was existed in the viral stock.

To deeply understand the genomic characteristics of SD20LH01, the full-length genome of SD20LH01 was obtained by PCR amplification and sequenced. The results showed that the proviral genome of SD20LH01 had typical characteristics of replication-competent avian retrovirus, with the structure described as 5’LTR-gag-pol-env-3’LTR. The *gag* and *pol* genes of SD20LH01 were relatively conserved compared with other reference strains, and there was no deletion or insertion mutation in the deduced amino acid. The difference between SD20LH01 and other ALV-K reference strains mainly located in *env* gene and U3 sequence (Chesters et al., 2002). SD20LH01 had 25 amino acid mutations in the envelop protein when compared with the prototype ALV-K strain JS11C1. These mutations might change the protein structure and affect its antigenic properties (Weber and Schaffner, 1985; Gao et al., 2015; Chen et al., 2020). As for U3 sequence, the homology between SD20LH01 and JS11C1 was only 51.9%, while the U3 region of SD20LH01 was closer to that of endogenous ALV, or subgroup E ALV. Since U3 region contained transcription factor binding sites and played promoter roles in the viral replication, it could be speculated that SD20LH01 might have a weak replication ability. To confirm this speculation, SD20LH01, JS111C1, and ALV-J strain NX0101 were inoculated into DF-1 cells and replication dynamics were recorded. It found that the replication rate of SD20LH01 was significantly lower than that of JS11C1 and NX0101, while NX0101 had an obvious advantage in replication ability. Due to the low replication efficiency of SD20LH01, ALV-K was easy to be neglected during daily monitor in chicken flocks, and resulting in virus vertical transmission. This also reminded us that more measures should be taken to improve the detection sensitivity especially when perform ALV clearance in Chinese indigenous breeds.

In order to further analyze the pathogenicity of SD20LH01, SPF chickens were infected with SD20LH01 by yolk sac inoculation and intraperitoneal inoculation. These 2 inoculation methods imitated vertical infection and congenital transmission at low age, respectively. Since subgroup J ALV was the most infectious and pathogenic subgroup, NX0101 strain was also infected with 1-day-old chickens as positive control. The results showed that chickens inoculated with SD20LH01 intraperitoneally produced a mild viremia within 7 wk postinfection, while chickens inoculated through yolk sac produced a lifelong persistent viremia. The results also showed that body weight was inhibited in chickens infected with SD20LH01 through yolk sac, while intraperitoneally infected group seemed affected slightly. Since ALV could cause immunosuppression in chickens, then we asked whether SD20LH01 could inhibit the immune response of vaccination (Cui et al., 2006). Therefore, NDV and AIV-H9 inactivated vaccines were injected, and NDV and AIV-H9 titers were evaluated. The results showed that SD20LH01 infection by yolk sac decreased the NDV titer, while SD20LH01 infection both 2 ways could interfere with AIV-H9 humoral immune response. Although this inhibitory effect was weaker than which caused by ALV-J, it also should be paid serious attention. During our animal experiment, no clinical signs were observed. This might be related to the low virulence of SD20LH01, also, we could not exclude the possibility that it had a long latent period, which would be studied in the further research.

Above all, a ALV-K strain SD20LH01 was isolated in a Chinese indigenous breed Luhua chickens in this study. SD20LH01 had a weak replication capacity which might be related with its endogenous ALV-like U3 sequence. Infection with SD20LH01 in SPF chickens induced growth retardation and decreased immune response to vaccines. Considering the huge number of Chinese indigenous breeds and the complicated infection status of ALV in those flocks, it is necessary to take a more stringent monitoring measure to speed up the ALV clearance project in indigenous chickens in China.
